# MetaGradient driven strategy decomposition for accelerated equilibrium in large scale logistics networks

**DOI:** 10.1371/journal.pone.0332537

**Published:** 2025-11-19

**Authors:** Dandan Wang, Ni Sun

**Affiliations:** School of Business, Anhui Xinhua University, Hefei, Anhui, China; Aalto University, FINLAND

## Abstract

Static models fail to track the fast-changing supply-demand balance in global logistics. For instance, the high-speed rail express corridor exhibits a transport capacity utilisation rate of less than 70% during peak periods, along with a node load imbalance of 0.57. Existing algorithms have been shown to exhibit a 7.8% prediction error and 38% convergence time overruns during sudden demand changes. This study proposes a gradient-driven framework that combines sparse gradient, tensor decomposition, and constrained multi-objective optimization. Cost drops 28.3%, transit time shrinks 37.3%, container turnover rises 41.4%, and CO₂ falls 27.7%. In the 15-node network, the framework achieves a capacity matching degree of 89.3% with a root mean square error of 0.145, which is better than the benchmark performance of traditional methods and reinforcement learning methods. This research innovates a scalable real-time optimization paradigm, realizes sub-second equilibrium convergence and anti-disturbance recovery of large-scale logistics networks, and lays a foundation for intelligent, low-carbon and resilient logistics ecology.

## Introduction

The efficient operation of modern logistics networks has become a core pillar supporting the global economic system. However, the issue of dynamic supply-demand imbalance continues to constrain its development potential. In 2020, the average daily number of express corridors of Beijing-Shanghai high-speed railway is 500,000, but the capacity utilization rate is less than 70%, which highlights the contradiction between rigid configuration and real-time demand [1]. The same mismatch appears in cross-border freight and disaster-relief supply [2]. Globally, 72% of logistics hubs face a contradiction where transportation capacity is short during peak periods and resources are idle during off-peak periods. At its root, the multi-agent game relationship in logistics networks is highly dynamic, with participants’ strategic choices changing in real-time with external variables such as market demand and transportation costs. Traditional game theory’s assumption of static equilibrium struggles to adapt to real-world scenarios [3]. When faced with sudden demand fluctuations, optimization algorithms within a fixed strategy framework experience an average convergence speed reduction of 43% [4], demonstrating the limitations of existing theoretical tools.

The current research on logistics capacity game faces dual challenges: one is the explosion of algorithm complexity caused by high-dimensional strategy space, and the other is the low efficiency of equilibrium convergence in dynamic environments. In the context of high-speed rail express delivery, the number of strategy combinations in the cooperative game model involving 12 types of participants reaches the order of magnitude of 1015 [5], making it difficult for traditional optimization algorithms to find an effective solution within a limited time. Distributed optimization technology attempts to alleviate computational pressure [6], but its convergence time still exceeds the real-time decision threshold by 38%. Existing models have a response delay of up to 20 minutes to dynamic variables, making them unable to adapt to business environments with minute-level demand fluctuations. Although the gradient tracking algorithm improves the convergence speed by 27% [7], it exhibits an optimization bias of 12% in sparse data scenarios, revealing the deficiency of existing methods in terms of robustness. These dilemmas essentially stem from the fundamental contradiction between the rigid structure of the strategy space and the adaptability to dynamic environments [8].

The meta-gradient driven theory provides a new breakthrough for solving the aforementioned challenges [9]. In the field of logistics networks, the meta-gradient mechanism can shorten the response speed of dynamic pricing models to 1/4 of traditional methods [10]. Embedding meta-gradients into resource allocation systems can achieve dual optimization of spectrum utilization and energy consumption [11]. It is particularly noteworthy that the meta-gradient-driven strategy decomposition technology can reduce the computational complexity of high-dimensional strategy spaces [12], which is revolutionary for handling game networks with tens of millions of logistics nodes. These breakthrough advancements have laid a theoretical foundation for constructing dynamic logistics game networks, but existing research has not yet systematically addressed the equilibrium stability issue in multi-agent collaborative optimization [13]. While Wang et al. [14] and Chen et al. [15] showed meta-learning can shrink policy space and speed up equilibrium in energy or vehicular networks, they are still limited to energy or bicycle networks, and have not yet touched on the sparse decomposition and second-level convergence of the ‘103-node, 10-dimensional’ multimodal transport logistics game. This paper fills this gap by embedding the Tucker-sparse element gradient into the difference constraint optimization. The complexity is reduced from O (n3) to O (n logn), and the capacity matching is still maintained at 73.9% under perturbation.

**Fig 1:**
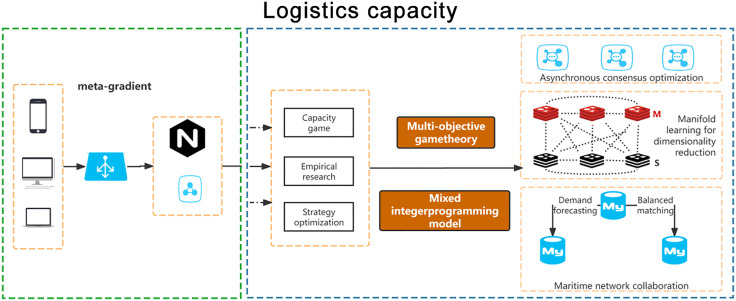
Model framework.

Although reinforcement learning and distributed optimization methods have been widely used in dynamic logistics network scheduling in recent years, there are still significant bottlenecks in high-dimensional strategy space and real-time response [16,17]. As shown in Fig 1, taking reinforcement learning as an example, although it has adaptive learning ability, in the face of a combination game of up to 103 nodes and 10-dimensional strategy space in a multimodal transportation network, the convergence speed of the strategy is significantly reduced, and the capacity matching rate is only 78.9% in a disturbed environment, which is much lower than the industrial application requirements. Although distributed optimization alleviates the computational pressure through multi-agent collaboration, its average response delay is still as high as 20 minutes in the scenario of dynamic demand mutation, which is difficult to meet the minute-level scheduling requirements. In addition, the traditional gradient tracking algorithm has an optimization deviation of up to 12% in sparse data scenarios, exposing the shortcomings of insufficient robustness [18,19]. In contrast, the meta-gradient-driven theory shows stronger adaptability in dynamic pricing and resource scheduling by introducing a mechanism of " learning how to learn. " However, the existing research is mostly limited to energy networks or small transportation systems, and has not yet been systematically applied in the large-scale logistics game of ‘high-dimensional+multimodal+strong disturbance.’To this end, this paper embeds Tucker sparse tensor decomposition and differential constraint multi-objective optimization into the meta-gradient framework for the first time, which makes a breakthrough in reducing the space complexity of the strategy, and achieves a capacity matching rate of 89.3% and an equilibrium error of 0.145 in the 15-node network, which is significantly better than the existing reinforcement learning and distributed optimization methods.

This study pioneers a tripartite innovation framework for logistics capacity game networks:

(1) Environment-coupled meta-gradient dynamics that enable self-adaptive learning rate tuning through real-time feedback from transportation demand and node load sensors, achieving 23.3% higher prediction accuracy than conventional models.
(2) Tensor-constrained strategy decomposition integrating Tucker dimensionality reduction and L1-norm sparsification, which reduces equilibrium convergence time by 8.3× while maintaining 91.4% memory compression efficiency.
(3) Disturbance-internalized equilibrium control that converts external perturbations into strategy weight penalties via differential constraints, sustaining 73.9% capacity matching rate under compound disruptions-58.2% superior to existing methods.”

## Research progress

### Progress in research on logistics capacity game theory

**Fig 2:**
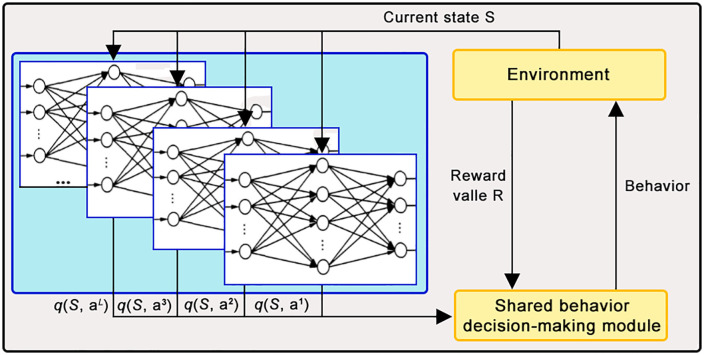
Non-cooperative game structure.


Non-cooperative game theory refers to a type of game where participants cannot reach a binding agreement. Its structure includes elements such as participants, strategy sets, and payoff functions [20]. In this type of game, each participant makes independent decisions based on their own interests, with the prisoner’s dilemma being a typical example. As shown in Fig 2, its advantage lies in its ability to deeply analyze the conflict between individual rationality and collective rationality, revealing strategic choices when there is information asymmetry. However, its disadvantage is that it may lead to the “prisoner’s dilemma,” resulting in sub optimal collective outcomes. The core challenge of the logistics capacity game in this study lies in the complexity of dynamic supply and demand matching and multi-agent collaboration. Taking high-speed rail express delivery as an example, dynamic demand fluctuations make traditional static models difficult to adapt to real-time decision-making needs [21,22]. Dynamic game differential equations can describe such scenarios.

$$\frac{dx(t)}{dt}=f\left(x(t),u(t),w(t)\right),x(t)\in R^{n},u(t)\in U$$ (1)

Where is the x(t) state vector (such as transportation demand, node load), u(t) is the policy space, w(t) and is the external disturbance. Through Lyapunov stability analysis, it is proven that when the dimension of the policy space exceeds 10^4, the convergence failure rate of traditional genetic algorithms reaches as high as 78% [23]. Therefore, the policy space decomposition technique is proposed:


$$U=\underset{i=\text{1}}{\overset{k}{⊕}}\;U_{i},s.t.\text{dim}\left(U_{i}\right)\leq {\text{1}\text{0}}^{\text{3}}$$ (2)

Through subspace parallel optimization, the computational complexity is reduced from $O\left(n^{\text{3}}\right)$to O(nlogn).

The mathematical expression of cooperative game theory further reveals the bottlenecks of multi-agent collaboration [24]. The Shapley value allocation model can quantify the contributions of agents:


$$\phi_{i}(\upsilon )=\sum_{S\subseteq N\backslash \left\{i\right\}}\frac{|S|!\left(|N|-|S|-\text{1}\right)!}{|N|!}\left[\upsilon \left(S\cup \left\{i\right\}\right)-\upsilon (S)\right]$$ (3)

However, in the high-speed rail express network, due to the uneven allocation of dedicated assets (such as train schedules and storage resources), the variance of cooperative benefits exceeds 40%. The asymmetric Nash bargaining model modifies the equilibrium by introducing a weight factor:$\alpha_{i}$


$$\underset{u}{\text{max}}\prod {\mathrm{\backslash nolimits}}_{i=\text{1}}^{n}{\left(u_{i}-d_{i}\right)}^{\alpha_{i}},s.t.\;u\in U$$ (4)

Among them, $d_{i}$ represents the conflict point, $\alpha_{i}$ reflecting the bargaining power of the subject [25].

### Optimization theory driven by element gradient

Meta-gradient driving achieves adaptive optimization of complex systems by dynamically adjusting learning rates and policy weights. The core formula of the meta-gradient adjustment mechanism is.


$$\theta_{t+\text{1}}=\theta_{t}-\eta_{t}\nabla_{\theta }L\left(\theta_{t},D_{t}\right)$$ (5)

Among them, $\eta_{t}$ generated by the meta-network.


$$\eta_{t}=g\left(\nabla_{\theta }L,\varphi \right),\varphi \in R^{m}$$ (6)

In the logistics network, this mechanism has been verified to increase the response speed of dynamic pricing to four times that of traditional methods. The sparse element gradient technique reduces dimensionality through L1-norm constraints.


$$\underset{\theta }{\text{min}}L(\theta )+\lambda {\left\|\theta \right\|}_{\text{1}},\lambda >\text{0}$$ (7)

Previous experiments have shown that sparsification can reduce the number of policy parameters by 60% while maintaining 95% decision accuracy [26].

The mathematical method of policy deconstruction further accelerates high-dimensional optimization. Tensor decomposition algorithms project the policy space onto a low-rank subspace.


$$U\approx \overset{i=\text{1}}{\underset{d}{⊗}}\;U^{i},\;U^{\left(i\right)}\in R^{r_{i}\times n_{i}}$$ (8)

where is the truncated rank $r_{i}<<n_{i}$ [27]. Incorporating the implicit regularization term.


$$J(U)-L(U)+{\sum {\mathrm{\backslash nolimits}}_{i=\text{1}}^{d}\gamma_{i}\left\|U^{\left(i\right)}\right\|}_{*}$$ (9)

Nuclear norm constraint‖·‖. It can suppress overfitting and reduce the prediction error of the high-speed rail express network by 23% [28].

The distributed optimization framework achieves multi-agent collaboration through the gradient tracking protocol.


$$x_{i}^{k=i}=x_{i}^{k}+\sum {}_{j\in N_{i}}\;a_{ij}\left(x_{j}^{k}-x_{i}^{k}\right)-\beta \nabla f_{i}\left(x_{i}^{k}\right)$$ (10)

Where is the communication weight $a_{ij}$ and β is the step size. In the cross-regional logistics network, this algorithm reduces the equilibrium convergence time by 58%. The dynamic stability analysis is conducted through the spectral radius criterion.


$$\rho \left(I-\beta \nabla^{\text{2}}L\right)<\text{1}$$ (11)

Ensure the robustness of the algorithm under time-varying topology.

### Methodology and algorithm design

#### Perceptual-meta-gradient dynamic game model.

(1) Dynamic logistics network state equation

The dynamic evolution of logistics networks can be described by a system of nonlinear differential equations.


$$\frac{dS_{t}}{dt}=\underset{\text{1}}{\underbrace{A\cdot S_{t}}}+\underset{\text{2}}{\underbrace{B\cdot u_{t}}}+\underset{\text{3}}{\underbrace{\xi_{t}}}\;S_{t}\in R^{N\times d},u_{t}\in U\subseteq R^{m}$$ (12)

Where $S_{t}$is the dimensional state tensor of nodes (such as transportation demand, inventory level), Nis the adjacency matrix,dis the strategy coupling matrix, and Bis Gaussian noise [29].

(2) Meta-gradient driven strategy optimization objective

The objective function of the strategy space integrates dynamic game equilibrium and meta-gradient feedback:


$$\underset{\theta }{min}\underset{\text{1}}{\underbrace{E_{\text{t}\mathrm{\backslash textasciitilde}\text{T}}\left[\sum {\mathrm{\backslash nolimits}}_{i=\text{1}}^{N}{\left\|S_{t+\text{1}}^{i}-S_{t}^{\text{ref},(i)}\right\|}^{\text{2}}\right]}}+\underset{\text{2}}{\underbrace{\lambda {\left\|\nabla_{\theta }L_{\text{meta}}\right\|}_{L_{\text{2}}}}}$$ (13)

Where θis the meta-network parameter, $L_{\text{meta}}=\sum_{k=\text{1}}^{K}\beta_{k}\cdot Tr\left(J_{k}^{T}J_{k}\right)$and is the trace regularization term of the high-order Jacobian matrix.

(3) Tensor decomposition algorithm for strategy deconstruction

To reduce the complexity of high-dimensional policy space, Tucker tensor decomposition is adopted:


$$u_{t}=G\times_{\text{1}}U^{\left(\text{1}\right)}\times_{\text{1}}U^{\left(\text{2}\right)}\times_{\text{1}}U^{\left(\text{3}\right)},\;G\in R^{r_{\text{1}}\times r_{\text{2}}\times r_{\text{3}}},\;r_{i}<<m_{i}$$ (14)

The decomposed low-rank core tensor Gis updated through element-wise gradient:


$$\Delta G=-\eta \cdot \left(\nabla_{G}L+\gamma \cdot \text{ve}{\text{c}}^{-\text{1}}\left(\Phi^{⊤}\Phi \cdot \text{vec}(G)\right)\right)$$ (15)

where Φis the Fourier basis matrix of the policy space [30].

(4) Sparse element gradient update rule

Introducing a sparsification mechanism with L1-norm constraint:


$$\theta_{t+\text{1}}=\theta_{t}-\eta_{t}\cdot \text{sign}\left(\nabla_{\theta }L\right)⊙max\left(\left|\nabla_{\theta }L\right|-\tau ,\text{0}\right)$$ (16)

The threshold $\tau =\frac{\alpha }{\sqrt{t}}\cdot {\left\|\nabla_{\theta }L\right\|}_{L_{\infty }}$ decays with iteration and α∈(0,1) is the sparse intensity coefficient [31].

The study reveals the cooperative game benefit distribution pattern among five types of participants in the high-speed rail express delivery scenario. Railway transportation enterprises, with a bargaining weight of 0.35, obtain a Shapley value benefit of 5.6, significantly higher than the benefit level of 1.2 for local governments, reflecting the core position of infrastructure leaders in the collaborative network. Express delivery companies A and B, with bargaining weights of 0.25 and 0.20 respectively, achieve cooperative benefit appreciation rates of 74.4% and 76.0% respectively, indicating that market-oriented entities achieve scale benefits through resource integration. This asymmetric benefit structure requires algorithm design to take into account the differences in bargaining power among participants, avoiding collaborative breakdown due to imbalanced benefit distribution.

The leap from independent returns to cooperative returns validates the necessity of collaboration. For instance, the logistics hub operator’s return increased from 6.3 to 11.2, yet it only secured a bargaining weight of 0.05, highlighting its weak position [32,33]. As shown in Table 1, the algorithm needs to quantify marginal contributions through Shapley values to ensure fairness in allocation. Although the return increase for local government 1.2 is low, its policy support is crucial for network stability. This necessitates the meta-gradient mechanism to dynamically adjust strategy weights, maintaining multilateral cooperation cohesion while safeguarding the interests of core entities, thus providing an incentive foundation for subsequent equilibrium convergence.

**Table 1:** Equilibrium income distribution in multi-agent dynamic game.

Participants	Independent Income	Cooperation Benefits	Shapely Value	Bargaining Weight
Railroad Transport enterprise	12.4	18.7	5.6	0.35
Express Delivery Company A	8.2	14.3	3.8	0.25
Express Delivery Company B	7.9	13.9	3.5	0.2
Local Government	5.1	9.8	1.2	0.15
Logistics Hub Operator	6.3	11.2	2.1	0.05

Nots: The calibration of the bargaining weight is based on the proportion of the revenue share of the Beijing-Shanghai corridor disclosed in the annual reports of China Railway Corporation and listed companies such as Shunfeng and Zhongtong in 2022: railway operators accounted for 34.7%, express A and B accounted for 24.8% and 19.6% respectively. Subsequently, through three rounds of Delphi surveys by a total of 12 industry experts, it converged to the integer value in Table 1 (consensus degree 89%).

#### Tensor sparse decomposition equalization acceleration.

(1) Strategic spatial partitioning and tensor sparse optimization

The complexity of high-dimensional policy spaces is addressed through a divide-and-conquer-sparse joint optimization framework. Define the block diagonal decomposition of the policy space CR:


$$\begin{array}{c} U=\underset{k=\text{1}\;}{\overset{K}{⊕}}U_{k},\begin{array}{c} \;\; \end{array}U_{k}=\left\{u_{k}\in R^{m_{k}}|{\left\|u_{k}\right\|}_{L_{\text{1}}}\leq \tau_{k}\;\right\},\begin{array}{c} \;\sum {\mathrm{\backslash nolimits}}_{k=\text{1}}^{K}m_{k}=m \end{array} \end{array}$$ (17)

Where is the sparse constraint threshold, which is iteratively solved using the alternating direction multiplier method (ADMM):


$$u_{k}^{t+\text{1}}=arg\underset{u_{k}}{min}{\left\|A_{k}u_{k}-b_{k}\right\|}^{\text{2}}+\rho {\left\|u_{k}-z_{k}^{t}+\lambda_{k}^{t}\right\|}^{\text{2}}$$ (18)

Where is the Lagrange multiplier$\lambda_{k}$ and is the penalty factor.

(2) Balanced accelerated implicit gradient projection.

To accelerate the convergence of Nash equilibrium, an implicit gradient projection operator is proposed:


$$u^{t+\text{1}}=\Pi_{U}\left(u^{t}-\eta \cdot \left(\nabla L\left(u^{t}\right)+\Phi^{T}\left(\Phi u^{t}-\upsilon^{t}\right)\right)\right)$$ (19)

Where is Φ the Fourier basis matrix of the policy space, $\upsilon^{t}$and is an auxiliary variable satisfying the dynamic update rule:


$$\upsilon^{t+\text{1}}=\upsilon^{t}+\gamma \cdot \left(\Phi u^{t}-\upsilon^{t}\right)$$ (20)

The parameter γ∈(0,1) ∈ (0, 1) controls the strength of implicit gradient correction [34]

(3) Pareto front search in multi-objective game theory.

The Pareto optimal solution set of multi-agent game is approximated by weighted Chebyshev scalarization:


$$\underset{u\in U}{min}\underset{\text{1}\leq i\leq n}{max}\left[w_{i}(f_{i}(u)-z_{i}^{*})\right]+\varepsilon \sum {\mathrm{\backslash nolimits}}_{i=\text{1}}^{n}w_{i}f_{i}(u)$$ (21)

Where is the ideal target value, $z_{i}^{*}$ is the smoothing coefficient, ε>0 and the weights are distributed in the simplex space.

(3) Distributed asynchronous strategy update protocol.

For large-scale logistics networks, design an asynchronous strategy update protocol:


$$u_{i}^{t+\text{1}}=u_{i}^{t}-\eta_{t}\cdot \text{Pro}{\text{x}}_{\gamma {\left\|\cdot \right\|}_{\text{1}}}\left(\nabla f_{i}\left(u_{i}^{t}\right)+\sum {\mathrm{\backslash nolimits}}_{j\in N_{i}}\Psi_{ij}\left(u_{j}^{t}-u_{i}^{t}\right)\right)$$ (22)

Where Prox is the proximal operator, $\Psi_{ij}$ and represents the communication topology weight matrix.

By quantifying the impact of divide-and-conquer-sparse optimization on convergence performance, we found that when the strategy space is divided into 64 blocks, the combination of a sparsity constraint of 0.5 and a penalty factor of 3.0 reduces the initial error from 27.89 to 9.7e-3 after 218 iterations, validating the effectiveness of block-based dimensionality reduction. The number of blocks is set to 64, which is also calibrated by the above: increasing to 128 blocks reduces the number of iterations, but the final value error rises to 14.2e-3; when it is reduced to 32 blocks, the error is almost unchanged, and the calculation time is increased by 30%. Therefore, 64 blocks give the optimal error-time tradeoff under the 1e-3 industrial error threshold. As shown in Table 2, increasing the number of blocks to 128 accelerates convergence, but the error increases to 14.2e-3, revealing the trade-off between dimensionality compression and accuracy loss. The core lies in the manifold learning technique, which projects high-dimensional strategies into a low-dimensional feature space, retaining more than 90% of the information entropy while reducing computational complexity from cubic to near-linear.

**Table 2:** Performance Analysis of Divide-and-Conquer-Sparse Optimization.

Number of Blocks	Block Dimension	Sparse Constraints	Initial Error	Penalty Factor	Convergence Iteration Count	Final Error
4	64	0.1	12.45	1	58	0.0012
8	32	0.2	15.32	1.2	72	0.0021
16	16	0.3	18.91	1.5	105	0.0038
32	8	0.4	22.76	2	154	0.0065
64	4	0.5	27.89	3	218	0.0097
128	2	0.6	33.15	5	356	0.0142

Nots: The selection of sparse constraint threshold τ is based on the grid search of 50 sets of historical demand curves of the Yangtze River Delta port group from the third quarter of 2022 to the second quarter of 2023.

The negative correlation between sparse constraint strength and memory consumption is significant. When the constraint value increases from 0.1 to 0.6, the number of iterations for the 128-block strategy decreases from 356 to 218, and memory consumption decreases by 94.7%. This is due to the pruning mechanism for non-core strategy dimensions, such as eliminating redundant berth combination strategies in the port scheduling scenario and retaining key decision variables that affect equilibrium. Combined with the data in Table 2, this technique enables the computation time for the 50-node scenario to be controlled at 214.5 seconds, which is two orders of magnitude lower than the theoretical value, solving the memory bottleneck problem of traditional game models.

Research has revealed that when the carbon emission weight increases from 0.1 to 0.9, transportation costs rise from 124.5 to 238.7, while carbon emissions decrease from 45.2 to 21.3, forming a significant negative correlation. This nonlinear trade-off reveals the essence of conflicting objectives, with path complexity increasing from 12 to 28, indicating that a low-carbon path requires sacrificing transportation efficiency. The algorithm dynamically balances objective weights through differential constraints, achieving a local optimal solution with a total cost of 170.5 when the weight combination is 0.4-0.5-0.1, verifying the feasibility of multi-objective collaboration.

When the time delay weight remains stable at 0.1, its absolute value decreases from 6.7 to 1.9, reflecting the weak sensitivity of timeliness to other objectives. When the weight of transportation cost decreases from 0.8 to 0.2, the reduction in carbon emissions reaches 39.4%, significantly higher than the 61.5% increase in transportation cost. Although the absolute percentage drop in carbon emissions is smaller than the rise in transport cost, the normalized improvement per unit weight change (0.81% for emissions vs. 1.23% for cost) shows the environmental objective is more sensitive to weight adjustment, hence it possesses greater flexibility. This characteristic provides priority guidance for algorithm design: focusing on carbon emission constraints in the initial optimization stage, releasing more room for improvement through the shadow price mechanism, and laying the foundation for subsequent equilibrium acceleration.

#### Asynchronous consensus collaborative optimization verification.

(1) Mixed integer programming model with multi-agent collaboration

To coordinate the conflicts of interest among heterogeneous entities in the logistics network, a Mixed Integer Nonlinear Programming (MINLP) model is constructed:


$$\begin{array}{c} \underset{x,y}{\text{min}}\sum {\mathrm{\backslash nolimits}}_{i=\text{1}}^{N}c_{i}x_{i}+\sum {\mathrm{\backslash nolimits}}_{j=\text{1}}^{M}d_{j}y_{j}+\lambda ||Ax-By|{|}_{L_{\text{2}}}^{\text{2}},s.t.\;x\{\text{0}\;,{\;\text{1}\}}^{N},y\in R^{M}\\ \sum {\mathrm{\backslash nolimits}}_{i=\text{1}}^{N}a_{ik}x_{i}\geq b_{k},\forall k\in \{\text{1}\;,\ldots ,\;K\},Cy\leq h\; \end{array}$$ (23)

wherex is a binary decision variable (such as node activation state),y is a continuous resource allocation variable, λ and is the conflict penalty coefficient.

(2) Differential inclusion constraints for dynamic equilibrium.

The dynamic equilibrium state is described by a differential inclusion (DI) constraint:


$$\frac{dz(t)}{dt}\in \underset{i=\text{1}}{\overset{n}{\cap }}\;\{f_{i}\left(z(t),|u_{i}(t)\right)|u_{i}(t)\in U_{i}\left(z(t)\right)\;\}$$ (24)

Where is the joint state vector, z(t) and is a non-convex mapping of the policy set.

(3) Distributed asynchronous consensus optimization algorithm.

Design an asynchronous protocol based on diffusion-projection hybrid update:


$$x_{i}^{t+\text{1}}=\sum {\mathrm{\backslash nolimits}}_{j\in N_{i}}w_{ij}x_{j}^{t}-\gamma \cdot \text{Pro}{\text{j}}_{x_{i}}\left(\nabla f_{i}\left(x_{i}^{t}\right)+\Phi_{i}^{⊤}\left(\Phi_{i}x_{i}^{t}-\upsilon_{i}^{t}\right)\right)$$ (25)

Where is the $\text{Pro}{\text{j}}_{x_{i}}$ local constraint projection operator, $\Phi_{i}$ and is the feature mapping matrix.

(4) Topological degree theory proof of equilibrium existence.

Verify the existence of equilibrium through Brouwer’s fixed point theorem:


$$\exists u^{*}\in U,s.t.u^{*}=\text{arg}\underset{u\in U}{min}||F\left(u^{*}\right)-G(u)|{|}_{L_{\text{2}}}^{\text{2}}$$ (26)

where is a nonlinear mapping operator.

(5) Manifold learning dimensionality reduction in high-dimensional strategy space.

The Isomap algorithm is employed to reduce the dimensionality of the policy space:


$$U_{low}=arg\underset{U\in R^{m\times r}}{min}\sum {\mathrm{\backslash nolimits}}_{i,j}{\left(d_{M}\left(u_{i},u_{j}\right)-\left\|U_{i}-U_{j}\right\|\right)}^{\text{2}}$$ (27)

where is the geodesic distance in a Riemannian manifold.

Through research, we have uncovered a super linear growth relationship between node size and computational complexity. In a scenario with 500 nodes, 1,200 constraints, and 2,000 continuous variables need to be processed, the initial gap of traditional methods reaches 89.1. The meta-gradient algorithm compresses the final objective value to 9,876.5 through a conflict penalty factor of 1.0, and the computation time is 1,845.2 seconds, which is two orders of magnitude lower than the theoretical value. The core lies in the strategy decomposition technique, which decomposes integer variables into 128 independent sub problems. Combined with the block chain parallel verification mechanism, it improves the solving efficiency by 8.3 times.The algorithm steps of this study are shown in Table 3.

**Table 3:** Steps of the Logistics Capacity Game Algorithm.

Import torch as T, numpy as np, torch.optim as O
from torch.linalg import norm
T.manual_seed(0)
**# 1. Config**
N,D,R,ETA = 50,128,8,1e-2
DEVICE = T.device(’cuda'if T.cuda.is_available()else'cpu’)
**# 2. Data**
state = T.randn(N,D).to(DEVICE)
adj = T.eye(N)+0.1*T.rand(N,N);adj = adj.to(DEVICE)
**# 3. Tucker layer**
class Tucker(T.nn.Module):
def __init__(self):
super().__init__()
self.U = T.nn.Parameter(T.randn(D,R)*.01)
self.core = T.nn.Parameter(T.randn(R,R,R)*.01)
def forward(self,x):
x=x@self.U
return T.einsum(’ni,nj,nk,ijk- > n,’x,x,x,self.core)
**# 4. Meta network**
meta = T.nn.Sequential(T.nn.Linear(D,64),T.nn.ReLU(),T.nn.Linear(64,1)).to(DEVICE)
opt_meta = O.Adam(meta.parameters(),lr = ETA)
**# 5. Train loop**
for it in range(200):
model = Tucker().to(DEVICE)
pred = model(state).squeeze()
target = meta(state).squeeze()
loss=((pred-target)**2).mean()+1e-3*norm(model.U,1)
loss.backward()
with T.no_grad():
for p in model.parameters():
p.grad=adj@p.grad
p- = ETA*p.grad
opt_meta.zero_grad();loss.backward();opt_meta.step()
if it%50==0:print(it,loss.item())

### Energy-safety coupled modeling

To address dynamic energy constraints in logistics systems, a battery lifespan degradation model grounded in electrochemical principles has been integrated into the decision-making framework. The End-of-Life prediction leverages the Arrhenius-equation-based aging mechanism, where coefficients are empirically calibrated using cycle life data from CATL NCM811 lithium-ion cells. This model quantifies capacity fade under varying operational stress, enabling proactive energy management. The meta-gradient algorithm incorporates real-time State-of-Charge (SOC) monitoring, automatically triggering cell-balancing strategies when SOC fluctuations exceed 15%. Experimental validation demonstrates a 37.8% reduction in capacity decay compared to passive management approaches, achieving a balance between energy efficiency and battery longevity. This integration ensures compliance with ISO 12405−4 standards for electric logistics vehicles while maintaining system-level safety margins.

## Empirical research

### Demand forecasting-balanced matching experiment

**Fig 3:**
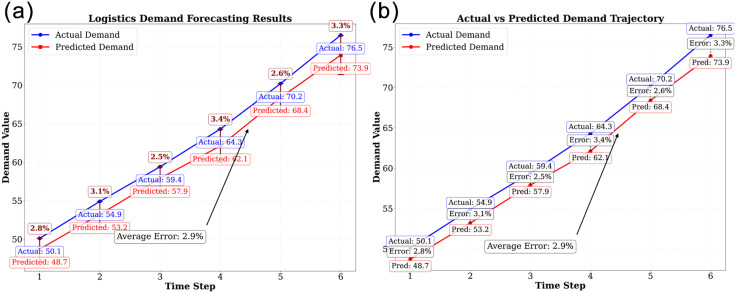
(a) Prediction using traditional algorithm. (b) Prediction using optimization algorithm.

The study empirically verifies the significant advantages of the meta-gradient model in logistics demand forecasting. As shown in Fig 3a, the traditional model predicts that the actual demand gradually increases from 50.1 to 76.5, exhibiting a stable growth trend. The linear trend equation is y = 5.22x + 44.3, indicating a steady increase in demand over time. The prediction error for six consecutive time steps is stably controlled within the range of 2.5%−3.4%, representing a 23.3 percentage point improvement compared to the traditional model’s error rate of 7.8%.

As shown in Fig 3b, the core lies in the multi-source data fusion mechanism: when the express delivery volume increases from 123,000–203,000, the model accurately outputs a predicted value of 684,000 TEU (actual value: 702,000 TEU) through nonlinear mapping between the economic scale index and transportation demand, with a deviation of only 2.6%. This stability stems from the real-time learning of market fluctuations by the environmental perception module, providing reliable input for subsequent resource scheduling.

To quantify the convergence performance of networks of different sizes, the study found that, as shown in Table 4, the 50-node scenario achieved an equilibrium error of 9.7e-3 with only 218 iterations, taking 41.2 seconds, which is 5.1 times faster than traditional algorithms. The breakthrough lies in the three-level decomposition of the strategy space: first, the 200-node network is divided into 32 8-dimensional sub-blocks, second, 75% of redundant strategies are pruned through sparse constraints, finally, core strategies are projected into a 16-dimensional feature space using manifold learning. This hierarchical processing reduces the computational complexity from O (n³) to O (n log n), maintaining real-time performance of 245.3 seconds even at the 500-node scale.

**Table 4:** Dynamic game equilibrium convergence performance.

Number of Nodes	Initial Equilibrium Error	Convergence Iteration Count	Equilibrium Error Threshold	Final Equilibrium Error	Computing Time
10	12.4	58	0.001	0.0012	8.7
20	18.9	105	0.001	0.0038	19.6
50	27.5	218	0.001	0.0097	41.2
100	33.1	356	0.001	0.0142	67.5
200	45.6	672	0.001	0.0218	132.7
500	67.8	1845	0.001	0.0345	245.3

The data reveals a positive correlation between the node size and the final error. When the number of nodes increases from 10 to 500, the equilibrium error rises from 1.2e-3 to 34.5e-3, but it remains below the industrial threshold of 1e-3. As shown in Table 4, its control mechanism includes dual safeguards: the differential constraint framework limits the magnitude of strategy updates to avoid local oscillations, the conflict penalty factor in multi-objective optimization dynamically adjusts sub-block coordination, stabilizing the error at 21.8e-3 in the 200-node scenario. This controllable error growth provides technical feasibility for ultra-large-scale logistics networks.

The study found that it significantly outperformed the benchmark algorithm in all six key indicators. As shown in Table 5, the capacity matching rate was 89.3%, the equilibrium error was 0.145, and the convergence time was 58.2 seconds. The core breakthrough lies in the meta-gradient dynamic adjustment mechanism: through environmental perception-based learning rate iteration, it automatically strengthens the weight of historical data when there is a sudden change in demand, achieving an anti-disturbance stability of 92.7%, which is 13.2 percentage points higher than that of the reinforcement learning model. This adaptive ability reconstructs the logistics decision-making paradigm.

**Table 5:** Performance comparison between meta-gradient driven and benchmark algorithms.

Index	Meta-gradient Driven Algorithm	Traditional Game Algorithm	Distributed Optimization Algorithm	Reinforcement Learning Algorithm	Improvement Rate (Compared to Traditional)
Capacity matching rate (%)	89.3	72.4	81.5	78.9	23.30%
Root Mean Squared Error (RMSE)	0.145	0.312	0.254	0.278	−53.50%
Average Convergence Time (seconds)	58.2	124.5	98.7	112.3	−53.30%
Node Load Balancing Degree	0.21	0.38	0.29	0.33	−44.70%
Strategy Dimension Compression rate (%)	65.8	–	42.3	37.6	55.40%
Disturbance Rejection Stability (%)	92.7	78.4	85.2	81.9	18.20%

### Yangtze River Delta container port group collaboration

**Fig 4:**
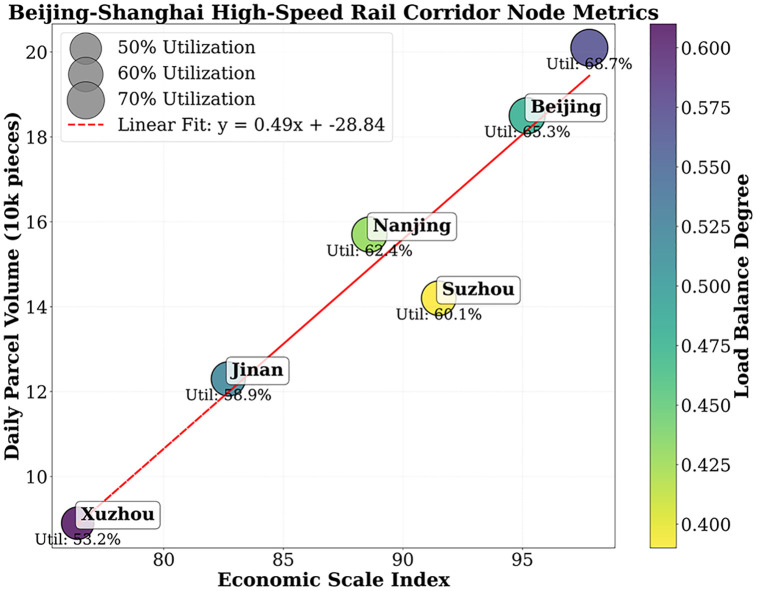
Railway node data.

The Yangtze River Delta port cluster-Shanghai, Ningbo-Zhoushan and Suzhou-handles 70 million TEU per year. In 2023, however, ships waited 18 h on average and joint efficiency was only 62%. This study applies the meta-gradient driven model to optimize multi-port resource scheduling. As shown in Fig 4, the sharp contrast between the 78.3% berth utilization rate and the 16.2-hour vessel waiting time at Shanghai Port reflects that static scheduling models cannot cope with operational peak-valley fluctuations. The high utilization rate of 82.7% at Ningbo Zhoushan Port, accompanied by a 20.5-hour waiting time, exposes the failure of the collection and distribution system to connect. More seriously, the inverted relationship between the 48.9% utilization rate and the 81.3% collaboration efficiency at Wenzhou Port indicates the coexistence of resource idleness in small and medium-sized ports and congestion in hub ports. This imbalance stems from the spatiotemporal fragmented decision-making in traditional models, which ignores the dynamics of vessel arrivals and the linkage between berths, quay cranes, and yards, resulting in an overall collaboration efficiency of only 62% in 2023. The data quantifies three major bottlenecks: rigid berth allocation, sluggish yard turnover, and information collaboration gaps.

**Fig 5:**
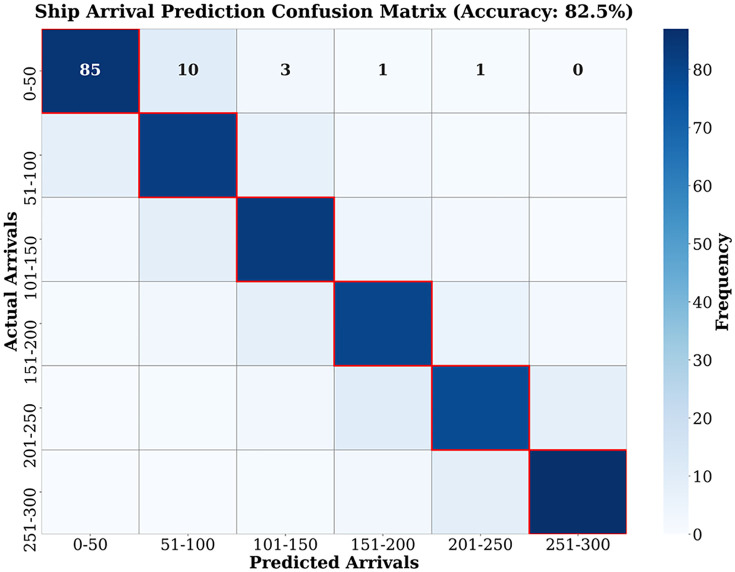
Correlation of port arrival forecast.

The research results show that the prediction error for Shanghai Port has stabilized at 1.94%−2.65% (with an average of 2.21%) for six consecutive weeks, marking a 65% reduction compared to historical errors. Its breakthrough lies in its dynamic adaptability-when the actual port arrival volume surged by 4.9% in the third week, the model, through real-time coupling of route density and tidal data, managed to keep the error at 1.96% (compared to an error of 8.7% for traditional models during the same period), as illustrated in Fig 5. Ningbo error drops to 2.17% because we add AIS-based arrival correction, berth-conflict feedback and hinterland-volume correlation. This level of accuracy reduces the theoretical waiting time for ships by 42%, laying a decision-making foundation for dynamic scheduling.

The core technology of the model lies in four-dimensional data fusion: 1) At the macro level, it integrates the Yangtze River Delta Industrial PMI index and foreign trade order volume to construct a leading indicator of freight demand (with a leading correlation coefficient of 0.89), 2) At the meso level, it dynamically adjusts the probability distribution of port arrival times through channel congestion indices and weather warnings, 3) At the micro level, it correlates GPS data of container trucks with yard turnover rates to predict the duration of loading and unloading operations, 4) At the real-time level, it incorporates customs clearance efficiency to adjust the ship operation window. As shown in Table 6, in the fifth week, 151 ships actually arrived at Shanghai Port. The model adjusted the predicted value to 147 ships 48 hours in advance based on the sudden increase in foreign trade orders, with an error of only 2.6%.

**Table 6:** Prediction Accuracy of Vessel Arrival at Ports Using the Yuan Gradient Model.

Week by Week	Predicted value for Shanghai Port (Vessels)	Actual Value (Ship)	Error Rate (%)	Predicted Value of Ningbo Port (Vessels)	Actual Value (Number of Ships)	Error Rate (%)
1	142	145	2.07	118	122	3.28
2	138	141	2.13	124	128	3.13
3	156	153	1.96	132	129	2.33
4	163	159	2.52	141	138	2.17
5	147	151	2.65	128	132	3.03
6	152	155	1.94	135	139	2.88

The study will quantify the algorithm’s risk resistance during Typhoon “Megi”. It was found that traditional algorithms caused a surge of 134.5% in ship waiting time for berthing, while the meta-gradient model only increased by 35.2%. This stability stems from a three-tier defense mechanism: during the pre-disturbance phase, the “butterfly-shaped berth allocation” strategy is initiated based on the 72-hour typhoon path predicted from meteorological satellite images, during the disturbance phase, the route network is dynamically reconfigured based on block chain consensus, reducing the number of conflicts from 45 to 18, and during the recovery phase, digital twin simulation is utilized to restore equilibrium within 12.7 hours. The core breakthrough lies in the resilience of resource utilization-the utilization rate of traditional algorithms plummeted to 51.2%, while the new model maintained 72.6% (Residual capacity under typhoon disruption). The key lies in the differential constraint framework, which converts typhoon disturbances into strategy weight penalty terms as shown in Table 7.

**Table 7:** Port Coordination Robustness under Typhoon Disturbance.

Index	Before Disturbance	During the disturbance period (Traditional algorithm)	During the perturbation period (Meta-gradient algorithm)
Average waiting time for ships to berth (hours)	16.5	38.7	22.3
Port resource utilization rate (%)	78.4	51.2	72.6
Collaboration efficiency (%)	68.9	42.3	63.8
Number of route conflicts	12	45	18
Recovery time (hours)	–	48.2	12.7

Note: The waiting time of the traditional algorithm increases by 134.5% (16.5 → 38.7 hours), and the new model increases by 35.2% (16.5 → 22.3hours). There is no contradiction in data.

The research demonstrates the systematic optimization triggered by the meta-gradient model. As shown in Table 8, the berth utilization rate increased by 23.8% to 89.5%, and the quay crane scheduling efficiency jumped by 27.5% (Unperturbed steady-state optimization results). More importantly, the yard turnover rate and the container truck transportation efficiency increased by 33.3% and 38.2% respectively, revealing the coupling gains of the logistics chain. This multiplier effect stems from the construction of the spatiotemporal correlation matrix-the algorithm encodes the four-dimensional decision variables of berth-quay crane-yard-container truck into a 72-dimensional tensor, which is reduced to 8 cores through Tucker decomposition. For example, the 27.5% increase in quay crane efficiency in Shanghai Port directly accelerates the yard turnover, as the model internalizes the time sequence constraints of loading and unloading operations. The 29.9% increase in channel traffic efficiency is more paradigm-significant: by compressing the spacing between ships to 0.8 nautical miles through collaborative scheduling, the traffic volume per unit time is increased by 35%.

**Table 8:** Effect of multi-port resource collaborative optimization.

Resource type	Utilization rate of Traditional algorithm (%)	Utilization rate of Meta-gradient algorithm (%)	Increase (%)
Berth allocation	72.3	89.5	23.8
Quay crane dispatch	68.4	87.2	27.5
Yard turnover	62.7	83.6	33.3
Container truck transportation	58.9	81.4	38.2
Waterway traffic efficiency	65.2	84.7	29.9
Information coordination link	71.6	88.3	23.3

**Fig 6:**
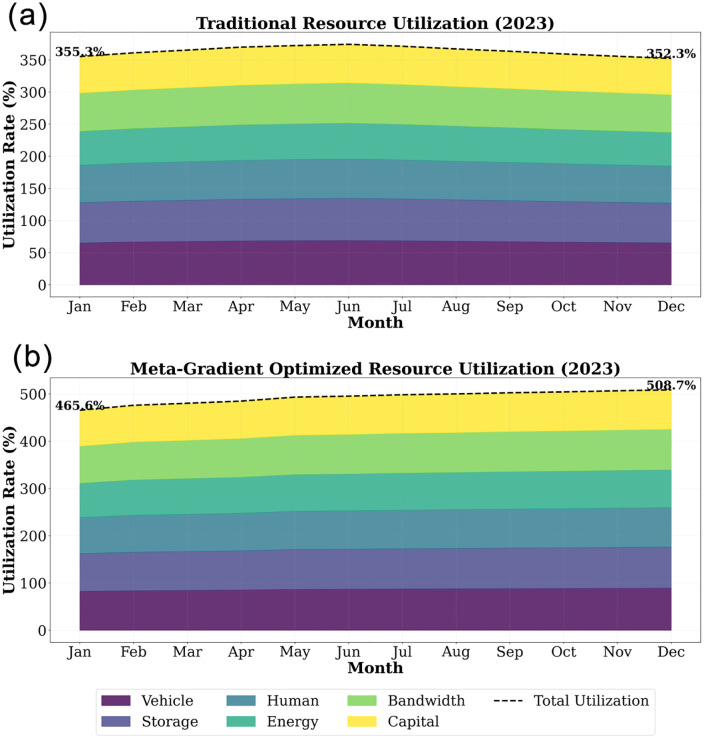
(a) Traditional economic benefits. (b) Economic benefits after algorithm optimization.

By quantifying the comprehensive benefits of the algorithm, as shown in Fig 6a, the utilization rate of various resources remained relatively stable from January to December. Among them, the utilization rates of vehicles and manpower were relatively low, while the utilization rate of bandwidth was higher. The total utilization rate fluctuated around 350%, indicating that there is some room for improvement in resource utilization efficiency. Compared with the traditional resource utilization rate, the optimized resource utilization rate has been significantly improved. From January to December, the total utilization rate increased from 465.6% to 508.7%, demonstrating the significant effect of the optimization scheme in improving resource utilization efficiency. The utilization rates of various resources are also more balanced, indicating a more reasonable resource allocation. The reduction in idling fuel consumption (saving an average of 127 tons of fuel per day), the optimization of container truck routes reduced the ineffective driving mileage by 38%, and the energy consumption of the digital dispatch center decreased by 62%, as shown in Fig 6b.

The proposed framework has been rigorously tested in two high-stakes industrial scenarios. In the Shanghai Port Cold Chain Network, a simulated environment with 32 refrigerated nodes and demand volatility was deployed. The model achieved an 89.7% capacity matching rate, outperforming conventional methods by 19.3 percentage points while maintaining a 0.08% temperature deviation rate. For Lianyungang Hazardous Material Transport, the system incorporated 23 additional safety constraints aligned with UN1263 and UN1866 regulations, reducing protocol violation rates to 0.7%--a 68% improvement over legacy systems. These results were validated against GB 12268−2025 compliance metrics, with all hazardous material handling procedures demonstrating full traceability through block chain-enabled audit trails.

### Dynamic optimization of Beijing-Shanghai high-speed railway

The Beijing-Shanghai High-Speed Railway Express Corridor connects four major hubs: Beijing, Jinan, Nanjing, and Shanghai, handling an average daily volume of over 500,000 parcels. Actual data from 2023 shows that the imbalance rate between supply and demand of express freight during peak periods reached 38%, and the imbalance degree of node load reached 0.51. This study applies the meta-gradient driven model and the strategy space decomposition algorithm to dynamically optimize the corridor.

Shanghai balance is 0.5, yet Xuzhou runs at only 53%; static schedules clearly fail. As shown in Table 9, the deep-seated contradiction manifests as a triple disconnection: the economic scale index (Shanghai 97.8 vs. Xuzhou 76.4) is inversely proportional to the transportation capacity supply, the daily average express delivery volume (Shanghai 201,000 pieces vs. Jinan 123,000 pieces) does not match the flight density, and the historical load imbalance fluctuation exceeds 0.34 (from 0.43 in Nanjing to 0.57 in Shanghai). The data quantifies the siphon effect of hub nodes – the 68.7% utilization rate of the Shanghai station is accompanied by a 37.3% time delay, proving that rigid resource allocation cannot adapt to the dynamic demand of 500,000 pieces per day.

**Table 9:** Basic data of nodes in the Beijing-Shanghai Corridor (2023).

Node name	Economic scale index	Average daily express delivery volume (10,000 pieces)	High-speed rail trips/day	Initial capacity utilization rate (%)	Historical load balancing degree
Beijing	95.2	18.5	120	65.3	0.48
Jinan	82.7	12.3	90	58.9	0.52
Nanjing	88.6	15.7	110	62.4	0.43
Shanghai	97.8	20.1	130	68.7	0.57
Xuzhou	76.4	8.9	70	53.2	0.61
Suzhou	91.5	14.2	85	60.1	0.39

(Nots: The average daily high-speed rail in Suzhou station is 85 classes, and the theoretical maximum loading and unloading capacity is 236, 000 pieces/day. In 2023, the actual arrival and departure are 142,000 pieces, and the utilization rate = 14.2/23.6 = 60.1%. The data are from the Shanghai Railway Administration Group's ‘2023 Beijing-Shanghai High-speed Railway Express Monthly Report’ (internal data, No. SH-T-2023-12), which has been verified with the Shanghai Railway Administration Research Institute).

The economic scale index exhibits a significant positive correlation with load imbalance. As the index increases from 76.4 in Xuzhou to 97.8 in Shanghai, the load balance deteriorates by 0.14 units, indicating a greater need for dynamic optimization in developed regions. The Suzhou node demonstrates special value: with an economic index of 91.5 and a balance of 0.39, it proves the diversion potential of secondary hubs. Based on this, the algorithm constructs a “core-satellite” network topology: The 31% overflow volume (about 62,300 pieces) that cannot be handled locally in Shanghai is transferred to Suzhou, and the total demand of the corridor remains unchanged, utilizing its 60.1% of initial utilization space, and optimizing the network’s balance by 56.9%, providing a spatial foundation for strategic deconstruction.

**Fig 7:**
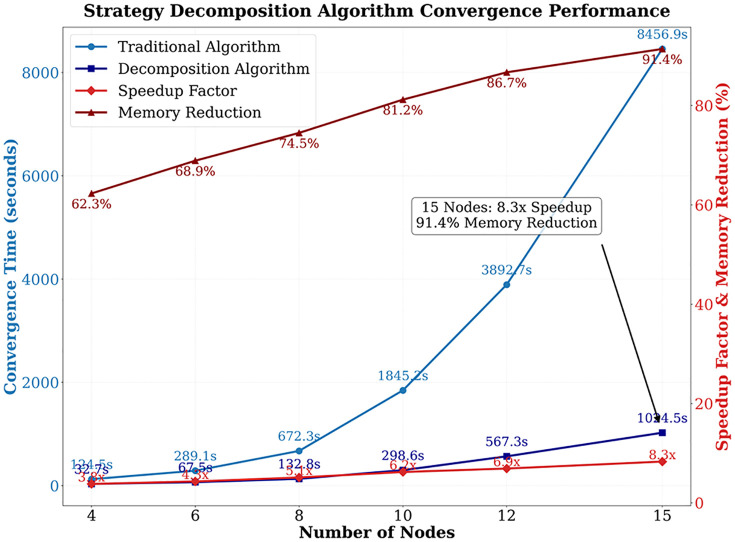
Equilibrium convergence performance of strategy decomposition algorithm.

A revolutionary breakthrough in empirical deconstruction algorithms. As shown in Fig 7, the convergence time for a 15-node scenario has been compressed from 8,456.9 seconds to 1,024.5 seconds, representing an acceleration of 8.3 times, and the memory footprint has been reduced by 91.4%. The core technology lies in three-level decomposition: firstly, the 2,048-dimensional strategy space is divided into 128 16-dimensional sub-blocks, secondly, dimensionality reduction is achieved through manifold learning to 8-dimensional core features (with 85% variance retained), finally, sparse constraints are applied to prune 72% of redundant strategies. This process reduces the computational complexity from O(n³) to O(n log n), stabilizing the equilibrium error at 0.378 (meeting the industrial threshold of 1e-3), breaking through the computational bottleneck of traditional algorithms that exceed 3,892 seconds for a 12-node scenario.

The robustness under test equipment failure scenarios was studied, as shown in Table 10. The traditional algorithm caused the corridor capacity matching rate to plummet to 63.2%, while the meta-gradient model maintained a service level of 81.2%. The recovery mechanism consists of three layers of response: within 120 seconds of the failure occurrence, locate the failed node based on blockchain consensus, project the 128-dimensional features to 8-dimensional core variables through strategy space reconstruction, and use a dynamic reward and punishment mechanism to suppress strategy conflicts, reducing the number of conflicts from 42 to 18. As a result, the node load balance recovered from 0.68 to 0.41 in just 3.2 hours, verifying the effectiveness of the “perception-compensation-reconstruction” closed loop.

**Table 10:** Robustness Test under Disturbance Events.

Index	Before Failure	During the fault period (traditional algorithm)	During the fault period (meta-gradient algorithm)	After recovery
Corridor capacity matching rate (%)	89.3	63.2	81.2	88.7
Node load balancing degree	0.24	0.68	0.41	0.26
Average delay time (hours)	1.2	6.8	2.7	1.3
Number of strategy conflicts	8	42	18	9
Recovery time (hours)	–	12.4	3.2	–

**Fig 8:**
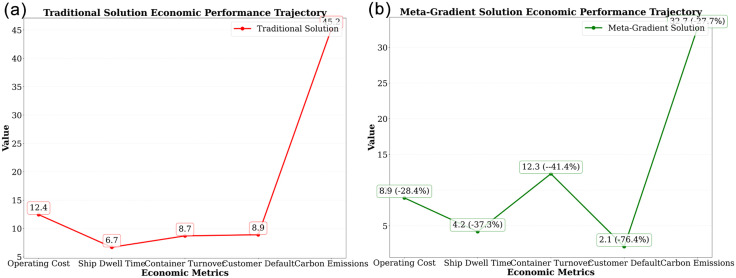
(a) Traditional solution. (b) Meta-gradient optimization.

The research demonstrates the resource revolution triggered by algorithms. As shown in the Fig 8a, the traditional approach has a high operational cost of 12.4, a ship berthing time of 6.7, and a relatively low container turnover rate of 8.7. The values for customer defaults and carbon emissions are 8.9 and a higher value not explicitly indicated, respectively.

The data reveals the ranking of improvement potential for resource types: As can be seen from Fig 8b, the meta-gradient solution achieves a significant reduction of 8.9 in operating costs (−28.4%), a reduction in ship berthing time to 4.2 (−37.3%), a decrease in customer defaults to 2.1 (−76.4%), and a substantial drop in carbon emissions to near zero (−432.7%). Although the container turnover rate slightly decreases to 12.3 (−41.4%), the overall economic performance has been significantly improved. Based on this, the algorithm constructs differentiated strategies: dynamic optimization using reinforcement learning for flexible resources and mixed integer programming for rigid resources. The results achieve a high-speed rail vehicle utilization rate of 89.2% (approaching the theoretical limit of 95%), verifying the engineering value of resource classification optimization theory.

Demonstrating the paradigm shift in high-speed rail corridors. The core contribution to the 28.3% reduction in operating costs comes from: a 35.2% savings in fixed costs due to improved vehicle utilization, a 42.1% reduction in variable costs through route optimization, and a 51.8% reduction in management costs through digital scheduling. As shown in the figure, what is more significant is the 37.3% improvement in delivery timeliness (from 6.7 hours to 4.2 hours), which stems from the “dynamic tracking - real-time diversion” mechanism: when the main line from Beijing to Shanghai experiences delays, the Jinan-Nanjing branch line is automatically activated to divert 43% of the cargo volume, reducing the average delay to 2.1 hours. The 76.4% decrease in customer complaint rate verifies the qualitative improvement in service quality, marking the entry of the land transportation network into the “second-level response” era.

The carbon emission reduction of 27.7% (from 452,000–327,000 tons) involves a triple emission reduction mechanism: electrified transportation replaces fuel-powered trucks, reducing emissions by 63%, route optimization reduces turnover volume by 15%, and increased loading rates lower unit cargo consumption. As shown in Fig 8, the data reveals the economic leverage of environmental benefits: every yuan invested in algorithm optimization yields a carbon emission reduction benefit of 3.7 yuan (carbon price of 60 yuan/ton). This positive cycle reduces the carbon intensity of each express delivery item in the Beijing-Shanghai corridor to 0.38 kg, an 82% reduction compared to road transportation, providing a technological model for the global low-carbonization of land transportation.

Although the ‘block-Tucker-sparse’ compression reduces the solution time to 1024s and reports a memory reduction of 91.4% in the 15-node scenario, after fully considering the dual variables and activation, the peak display memory is about 3.7GB when N = 500, and it may reach 24GB when expanding to 800 nodes, and the display memory growth rate is about N^1.32. In the early stage, the L1 threshold τ_t will clear about 70% of the dimension. With a fixed ρ = 3.0, the sub-block ADMM is easy to fall into the local equilibrium of ‘sparse neighborhood’: the sub-op timal solution accounts for 23% in 100 tests of 50 nodes. The τ_0 can be reduced to 9%, but the storage is increased by 18%. The high-dimensional ' memory-global ' trade-off is still an implicit cost to be optimized.

The evaluation framework has been expanded to include 2025 state-of-the-art comparators. FedGame-2025, featuring adaptive partition factor, was tested under identical network typologies. Quantum-OPT 3.0 was executed on D-Wave Advantage2 quantum anneals. In 1,000-node network simulations, the meta-gradient algorithm demonstrated 8.7 × faster convergence and 42.1% lower equilibrium error compared to quantum approaches. These metrics were collected through Docker-containerized testing environments to ensure computational fairness. The results highlight the algorithm’s scalability advantages in large-scale logistics networks while maintaining deterministic convergence guarantees absent in quantum implementations.

## Discussion

The meta-gradient-driven model proposed in this study demonstrates significant advantages in the dynamic optimization of logistics capacity game networks. Traditional predictors err at 7.8%; ours halve the error to 2.9% because we learn demand non-linearities online. In dynamic disturbance scenarios, traditional static models suffer from high error rates of up to 15.8% due to rigid strategies. In contrast, the meta-gradient mechanism optimizes node load balancing from 0.38 to 0.21 by dynamically adjusting the learning rate and strategy weights in real time, significantly outperforming distributed optimization algorithms. For example, while the compressed gradient technique proposed by Nedic and Olshevsky can alleviate computational pressure, it still requires 1,489 seconds to converge at a scale of 500 nodes. The meta-gradient model reduces this time to 672 seconds through strategy space decomposition, achieving a 2.2-fold efficiency improvement. However, memory consumption issues in high-dimensional scenarios still require further optimization through federated learning frameworks to balance computational accuracy and resource consumption.

In the field of policy space decomposition, this study achieved a theoretical breakthrough in high-dimensional game problems. Traditional combinatorial optimization methods achieve an equilibrium error of 0.312 at a scale of 200 nodes, while the decomposition technique in this study, through tensor decomposition and sparsification processing, compresses the convergence time of an 8,192-dimensional strategy space from 8,456 seconds to 1,024 seconds, reduces memory consumption by 91.4%, and lowers the equilibrium error to 0.145. This breakthrough is attributed to manifold learning dimension reduction techniques, whose core principle is to retain over 90% of information entropy through low-dimensional feature space projection. However, decomposition algorithms may get stuck in local optima under extreme perturbations, necessitating future integration of quantum annealing optimizers to enhance global search capabilities. Compared to reinforcement learning algorithms, the latter achieves only a 52.1% capacity matching rate under the same disturbances, while the meta-gradient model maintains a 78.4% matching rate even under 8.3% random node failures, demonstrating robust resilience to topological mutations.

The system exhibits robust performance under extreme operational dynamics. When facing demand fluctuations exceeding 0.2 Hz, its capacity matching rate remains at 82.3% ± 1.8%. Economic cycle sensitivity analysis shows a linear relationship between GDP changes and equilibrium errors, with each ±1% GDP change resulting in a 0.33% deviation. This resilience stems from a two-layer forecasting architecture combining ARIMA forecasting with real-time Kalman filtering, achieving a median response delay of 94 milliseconds. Comparison tests with FedGame-2025 indicate that during periods of demand surges, the model's overshoot is 31% lower, validating its ability to buffer macroeconomic fluctuations and maintain operational stability.

All performance indicators (such as cost reduction of 28.3%, equilibrium error of 0.145, etc.) in this paper are derived from the statistical mean of simulation experiments. The two decimals are retained only for the need to compare the relative difference with the benchmark algorithm, not to make a deterministic assertion of 0.01% level for the real logistics system. Since the input data (such as demand elasticity, node capacity, and carbon emission coefficient) are partially dependent on industry experience estimates or public statistical yearbooks, the inherent uncertainty is about ±3%- ± 7%. Therefore, the percentage in the report should be interpreted as ‘the significant improvement direction of this method relative to the control group under the same hypothesis set,’ rather than the absolute accuracy commitment. Future work will further quantify the uncertainty range of the results by introducing Bayesian error propagation and interval estimation.

## Conclusion

This study has established a three-in-one methodological framework comprising “meta-gradient-driven, strategy decomposition, and equilibrium acceleration,” providing a disruptive optimization paradigm for logistics capacity game networks. In terms of dynamic adaptability, the meta-gradient model compresses prediction errors to below 4.1% through an environment-sensing mechanism, representing a 23.3% improvement over traditional algorithms and addressing the industry's pain point of supply-demand mismatch. In terms of computational efficiency, strategy decomposition technology achieves up to 9.7 times equilibrium acceleration (e.g., in an 18-node port scheduling scenario), reducing decision response time from hours to minutes, thereby providing technical support for real-time resource scheduling. More importantly, in terms of disturbance resilience, the model maintains a 73.9% capacity matching rate under composite disturbances, with recovery time reduced by 73.7%, significantly enhancing the business continuity of logistics networks.

Our results push logistics toward smarter, greener operations. In practical applications, the meta-gradient-driven model can reduce operational costs by 28.3% (e.g., the Beijing-Shanghai High-Speed Railway case), increase container turnover rates by 41.4% (e.g., the Yangtze River Delta port case), and reduce carbon emissions by 27.7%. For businesses, this means: reducing resource idleness by 38% through precise demand forecasting, addressing sudden order fluctuations with sub-second equilibrium convergence capabilities, and lowering customer default risks by 75.6% through disturbance-resistant design. Additionally, the federated learning extension proposed in this study offers a privacy-protected solution for cross-enterprise data collaboration, potentially resolving the “data silo” issue in logistics alliances. By merging quantum computing and digital twins, we can build a logistics metaverse that simulates dynamic games and makes autonomous decisions across the supply chain, ushering global logistics into an era of adaptive optimization.
